# Social network correlates of HIV risk-related behaviors among male migrants in China

**DOI:** 10.1186/s12889-017-4409-2

**Published:** 2017-05-17

**Authors:** Wenqing Wang, Kathryn E. Muessig

**Affiliations:** 1 School of Humanities and Social Sciences, Beijing Institute of Technology, 5 South Zhongguancun Street, Haidian District, Beijing, 100081 China; 2 Department of Health Behavior, Gillings School of Global Public Health, University of North Carolina at Chapel Hill, Chapel Hill, NC USA

**Keywords:** Social network, Commercial sex, Condom use, Male migrant, China

## Abstract

**Background:**

Significant domestic and global research has focused on HIV risk among China’s large internal migrant population. Much of this work takes an individual behavior approach while ignoring the critical role social networks play in shaping HIV risk.

**Methods:**

Based on past studies among migrant men in China of *yingchou* activities (activities that build and reinforce social networks such as eating, drinking alcohol and patronizing commercial sex), we constructed ego-centric networks for a sample of 385 male migrants recruited from multiple worksites in Beijing. We used a nested-model approach to examine the contribution of social network characteristics to HIV risk at both the variable and model levels.

**Results:**

As compared to an individual-level model, addition of social network variables significantly improved the fit of the models. Commercial sex norms and condom use norms of core *yingchou* networks were significantly associated with egos’ commercial sex and condom use respectively. The size of *yingchou* network was associated with egos’ commercial sex. The network models became more sensitive after network norm measures took into account the intimacy of network ties and allowed for egos’ uncertainty when reporting their alters’ sexual behaviors.

**Conclusion:**

Results suggest the importance of social network factors and core network members in HIV transmission and risk-reduction interventions for male migrants. Future studies could explore other important social networks among male migrants, consider the intimacy of network ties and egos’ uncertainty about alters’ situations in constructing network norms, and refine the measurement of network size and density.

## Background

China has one of the world’s largest and fastest growing internal migrant populuations, increasing from 144 million in 2000 to 253 million in 2014 [1, 2]. Globally, the significance of migration has long been recognized as contributing to the spread of HIV through the geographic movement of people and disease, increased vulnerability of migrants, and increased risk behaviors associated with migration [3–5]. In China, the majority of research on migrants has focused on rural-to-urban migrant populations because they constitute the majority of internal migrants (about 75%, [2]) and are considered a potential high-risk bridging population of HIV infection between urban and rural China [6, 7]. Two recent meta-analyses found that compared to the general Chinese population, rural-to-urban migrants are at greater risk for HIV infection and other sexually transmitted infections (STI) [7, 8].

To date, efforts to explain the HIV risks among China’s internal migrants have primarily focused on analyzing the attributes of individuals, including socio-demographic attributes (e.g. no stable sex partner [9]), psychological attributes (e.g. risk perceptions [10]), and relevant risk behaviors (e.g. alcohol drinking, [11]). To better understand the vulnerabilities of migrants to HIV, it is important to explore the social contexts in which HIV risk behaviors among migrants are shaped [12–14]. The importance of social networks is well-established in HIV research [15–17] and China migrant studies [18–20], respectively. However, in studies that involve *both* migrants and HIV risk, the role of “networks” tends to be applied as a sampling method [21, 22] rather than a level of analysis to understand social contexts and drivers of risk behaviors.

This study aims to characterize the associations of social network characteristics and HIV risk behaviors—specifically commercial sex utilization and condom use during commercial sex—among rural-to-urban migrant men in China. The first step in collecting social network data is to define network boundaries [23]. Commercial sex was selected because it helped us formulate a specific social network relevant to HIV risk among male migrants. Past studies show that commercial sex is an HIV-related behavior involved in a culturally specific way in network building activities among Chinese men in general [24, 25] and migrant men in particular [26, 27]. In China, both migrant and non-migrant men tend to seek commercial sex in the context of groups as a social activity rather than alone [28, 29]. Commercial sex in such contexts is usually embedded in networking activities which are called “*yingchou*” in Chinese. *Yingchou* activities often start with social game-playing and eating and drinking, and end with female entertainment or services (often including commercial sex). As a cultural strategy to build personal and professional networks and facilitate exchanges among potential business partners, *yingchou* mixes emotional and economic interactions with the goal of establishing a strong relational foundation for future business transactions [25–27]. Commercial sex plays an important role in this process because it helps men select reliable business partners by assessing each other’s self-control and business competence through their performances in sex consumption [25]. Group-based utilization of commercial sex also strengthens the emotional bonds between business partners through a shared sense of intimacy established through collective participation in an activity deemed deviant by mainstream society [24–28]. In a sense, commercial sex consumption in *yingchou* constitutes a rite of passage from “outsider” to “insider” [30] within a social network.

In past research, *yingchou* has been almost exclusively studied qualitatively from a dynamic perspective which highlights the process of networking activities [25–27]. We propose a new perspective which focuses on the structural characteristics of the social network that results from *yingchou* networking activities. Here we use “*yingchou* network” to denote an ego-centered network that emerges from *yingchou* activities. As a widely used instrument to study the relational environment surrounding individuals, an ego-centered network consists of a focal actor, termed “ego”, and a set of alters who have specific ties to the focal ego actor [31]. In the context of *yingchou* network for this study, the focal ego actors (henceforth referred to as “ego”) are the male migrants who completed the study survey; the “alters” are the men named by egos who have participated in *yingchou* activities with the ego over a certain period of time. Since commercial sex is usually embedded in *yingchou* activities, we hypothesized that “*yingchou* network” may be strongly associated with the likelihood of a male migrant to have commercial sex. As revealed by past research, y*ingchou* provides an environment in which restraints from traditional culture and society are temporarily relaxed and men feel more comfortable to communicate about commercial sex and condom use [27, 28]. These communications facilitate the development of peer norms concerning commercial sex and condom use [27, 28], which have significant influence on male clients’ condom use in commercial sex [29]. Therefore, we also hypothesized that condom use among *yingchou* network members would be associated with egos’ condom use in commercial sex.

## Methods

### Study site

The data were collected from July to August in 2014 among rural-to-urban migrants in Beijing, China. In this article, a migrant is defined as a person who lives in Beijing but has a rural *hukou* (official household registration status) outside of Beijing. Beijing has a population of 21.15 million, 38% (8.03 million) of whom are migrants [32].

### Participants and sampling

**Fig. 1:**
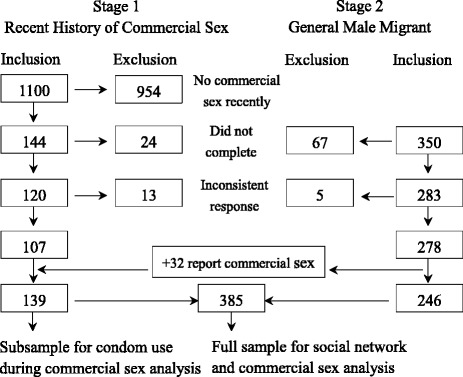
Sampling stages and data management

One urban district and one suburban district in Beijing were chosen as the sites for sampling. The inclusion criteria of participants were as follows: a) males aged 18 to 60 years old; b) registered as a rural resident outside of Beijing. The sampling was divided into two stages (Fig. 1). The first stage was to ensure sufficient recruitment of male migrants (>100) who had participated in commercial sex in the last year (“male migrant clients”). Convenience sampling was used to recruit male migrant clients. In places where migrants frequently gather - such as construction sites, factories, markets, and restaurants - interviewers approached men to confirm their migrant status, introduce the survey, and invite them to participate. The digital self-administered questionnaire was stored on an Internet server, and accessed and delivered through mobile phones or tablets connected to the Internet. The first part of the questionnaire was pre-selection questions to identify whether they had had commercial sex in the last year and confirm their age and migrant status. Eleven hundred migrants consented to participate in the survey, of whom 956 were excluded due to no commercial sex in the last year. Among the remaining 144 participants, 120 completed the full questionnaire. Thirteen participants were excluded from the dataset for final analysis because they provided inconsistent answers for three questions on commercial sex experience. Participants excluded by pre-selection (no recent commercial sex) received 10 RMB (about 1.5 USD) remuneration; participants who finished the survey received 50 RMB (about 8 USD); and participants who dropped out while taking the survey received 10–50 RMB based on their time spent on the survey.

In the second stage, the same sampling method was applied to recruit general male migrants who may or may not have had commercial sex in the last year. Three hundred and fifty participants were enrolled in the second-stage survey; 283 participants finished the survey; five participants were excluded from the dataset for final analysis because of inconsistent answers to three questions on commercial sex.

Thirty-two men recruited in the second stage reported commercial sex experience in the last year and were thus combined with the 107 participants from the first-stage sample to constitute the final dataset for condom use analysis (subsample, *n* = 139). Participants from both stages were combined as the final sample for commercial sex analysis (full sample, *n* = 385).

All procedures performed in this study were in accordance with the 1964 Helsinki declaration and its later amendments or comparable ethical standards. We obtained ethics approval from the ethics committee of the Beijing Institute of Technology.

## Measurement

### Demographic characteristics

Data on age, education level (i.e. primary school or below, junior high school, senior high/professional school, college or above), monthly income (less than 3000 yuan, 3000–5000 yuan; 5000–10,000 yuan; at least 10,000 yuan), employment (employed; self-employed or other), marital status, steady sex partner (yes, no), and frequency of *yingchou* in the last year (Question: “How frequently have you participated in *yingchou* in the last year for the purpose of business, work or making a living?” Answer choices: never, once or less per month, two to three times per month, once a week, at least twice per week) were collected.

### Perceptions of HIV/STI risk and commercial sex

Adapted from the scales used in an earlier study on HIV/STI risk behaviors and perceptions among rural-to-urban migrants in China [10], nine questions evaluated participants’ perceptions around commercial sex and HIV/STI risk. Two items were used to assess perceived vulnerability to HIV and STIs separately (the two items’ Cronbach’s Alpha, 0.93). Each response was coded on a five-point scale from “very unlikely to get infected” (1 point) to “very likely to get infected” (5 points). A composite score was calculated by adding responses of the two items (range 2–10), where higher composite scores indicate higher perceived vulnerability to HIV. Similarly, two items were used to assess perceived severity of getting infected with HIV/STIs (Cronbach’s Alpha, 0.84), and another two items were used to assess perceived necessity and effectiveness of condom use in commercial sex (Cronbach’s Alpha, 0.86). The three derivative variables (perceived vulnerability to HIV/STIs, perceived severity of being infected with HIV/STIs, and perceived necessity and effectiveness of condom use in commercial sex) were used in the analysis both of condom use and of commercial sex.

Three items were used to assess perceived social motivations and deterrents of commercial sex. Among them, two items measured desirable outcomes of commercial sex (the relief of loneliness, a symbol of social status) and one item measured an undesirable outcome of commercial sex (getting arrested by police). A composite score was calculated by adding responses of the three items (range 3–15; undesirable outcome scores were inverse coded; Cronbach’s Alpha, 0.82), with a higher score indicating higher perceived social motivations to engage in commercial sex. This fourth derivative perception variable was only used in the analysis of commercial sex.

A preliminary factor analysis (principal component analysis) of all nine perception items was performed to confirm the factor structure of the scales. The results showed that the nine items were clearly organized into four groups in common factor space, and that the emerging four key factors – perceived necessity and effectiveness of condom use in commercial sex (eigenvalue 2.56), perceived social motivations of commercial sex (eigenvalue 2.42), perceived vulnerability to HIV/STIs (eigenvalue 1.73), and perceived severity of being infected with HIV/STIs (eigenvalue 0.89) – corresponded to the four derivative variables used in this analysis.

### Size of full *yingchou* network

As stated above, the ego is the respondent who directly took part in the survey research, while the alters are men (named by the respondent) who have participated in *yingchou* activities over a certain period of time together with a certain respondent. The size of an ego’s *yingchou* network is the number of alters, excluding family members, who had participated in *yingchou* activities together with the respondent in the last year. Since it was very difficult for respondents to give an accurate number, we asked them to select an estimated number from four categories: 0–5, 6–10, 11–20, and >20.

### Size of core *yingchou* network

The size of a typical *yingchou* network was generally too large for feasibly describing each alter in detail (socio-demographic characteristics; the type, contact frequency, and closeness of alter-ego ties; the relationships among alters; risk behaviors, etc.). We therefore asked participants to report up to three alters who they considered most important to them. These alters constituted the “core *yingchou* network”. Although five-alter limits can also be used [33], we applied a three-alter limit for this study in order to recreate ego’s networks in detail (greater number of questions about each of an alter’s attributes and alter-alter relationships) while not posing a significant data-reporting burden on male migrant participants who have extremely limited time to contribute to research studies.

### Density of core *yingchou* network

Participants were asked whether the alters in their core *yingchou* networks knew each other. These responses were used to calculate the density of core *yingchou* networks as the proportion of total possible alter-alter ties that existed (possible score range: 0–1) [34]. This indicator was only relevant to the networks with two or three named alters.

### Norms of core *yingchou* network

The norms regarding commercial sex and condom use in participants’ core *yingchou* network were constructed to capture the popularity of commercial sex and condom use among network members. We measured each norm in two ways. The first way (corresponding to the analysis of Model 2 below) used the number of core network alters who were reported (by ego) as having the measured behavior. In the case of the commercial sex norm, the number of core network alters who had commercial sex in the last year was dichotomized into (a) no alter having commercial sex (assigned a value of 0) or (b) at least one alter having commercial sex (assigned a value of 1). With regard to condom use norm, the number of core network alters who used condoms consistently in commercial sex was dichotomized into (a) no alter using condoms consistently (assigned a value of 0) or (b) at least one alter using condoms consistently (assigned a value of 1).

The second approach for measuring norms (corresponding to the analysis of Model 3 below) allowed for more uncertainty in ego’s report of alters’ behaviors, assigned values to alters’ different behavioral statuses, and weighted the values of alters’ behaviors by the closeness of ego-alter ties. For the commercial sex norm, when asked whether alters had commercial sex in the last year, participants could report “yes”, “no”, or “not sure”, which were assigned values of 1, −1, and 0, respectively. For the condom use norm, egos similarly reported level of (un)certainty in their assessment of alters’ consistent condom use behaviors during commercial sex. Intimacy of alter-ego tie was measured with an 8-item scale (possible score range: 8–40; Cronbach’s Alpha 0.90, higher scores indicate higher degree of intimacy), which captured the length of time the ego knew an alter, the contact frequency between them in the last year, communicative intimacy (talking about intimate matters such as privacy, sex, HIV/STIs, and condom use), and behavioral intimacy (visiting entertainment establishments together). We computed a commercial sex norm score of an ego’s core *yingchou* network by multiplying each alter’s commercial sex status by his intimacy with ego and summing these products for a total score. Condom use norm scores were calculated through the same procedure.

### Commercial sex and condom use

Commercial sex experience was assessed by asking participants whether they had had commercial sex in the last year. Three differently worded questions (at the beginning and end of the survey) were used to improve the validity of data on commercial sex behavior. The response was dichotomized as “yes” or “no”. Survey respondents with inconsistent answers to these three questions were excluded from this data analysis to improve the overall data quality and validity.

Consistent condom use was assessed by asking participants whether they had used condoms during each instance of commercial sex in the last year. The response was also dichotomized as “yes” or “no”.

### Data analysis

The dependent variables in this analysis were commercial sex utilization and condom use in commercial sex. Independent variables included both individual-level variables (sociodemographic characteristics and risk perceptions) and network-level variables (network size, density, and norms). Multivariate logistic regression models were conducted to assess the relationship between independent variables and outcome variables. Nested models were employed to show the unique contribution of social network variables to the understanding of outcome variables. Among the three models, the first was an individual model including only individual-level characteristics. The second and third models added network variables to the individual model, each using different measures for network norms. Since the first model was nested in both models 2 and 3, we used a likelihood-ratio statistic (G^2^) to test whether the addition of network variables significantly improved the fit of the model [35]. The two network models were not nested, allowing determination of which model fit better (lower G^2^). However, it was not possible to determine whether the difference in fit between the models was statistically significant. SPSS 18.0 was used to conduct statistical analysis.

## Results

### Sociodemographic and network characteristics

Sociodemographic and network characteristics of participants are reported in Table 1. The full sample (*n* = 385) had a mean age of 31.6 years and was relatively well-educated (45.5% with an education above high school). Almost half of participants (46.2%) earned a monthly income of 5000 yuan (about 770 USD) or more. More than half were employed (59.0%), married (59.2%), had a steady sex partner (77.7%), participated in *yingchou* activities at least twice per month (56.1%), and had more than five alters in their full *yingchou* network (53.8%). The average density of core *yingcho*u network was 0.59 (SD = 0.34). Participants reported a total of 988 alters in their core *yingchou* networks. 25.8% (255/988) of these core *yingchou* network alters were reported having commercial sex in the last year. Among these reported alters, 52.5% (134/255) were reported to always use condoms during commercial sex in the last year.

**Table 1:** Sociodemographic and network characteristics of participants (*N* = 385)

Characteristic	N (%)
Age^a^	31.56 (9.09)
Education	
≤ High school	210 (54.55)
> High school	175 (45.45)
Monthly income	
< 5000 yuan	207 (53.77)
≥ 5000 yuan	178 (46.23)
Employment	
Employed	227 (59.96)
Self-employed or other	158 (41.04)
Marital status	
Married	228 (59.22)
Unmarried	157 (40.78)
Steady sex partner	
Yes	299 (77.66)
No	86 (22.34)
Networking frequency	
≥ Twice per month	216 (56.10)
< Twice per month	169 (43.90)
Consistent condom use in commercial sex (subsample, *N* = 139)	99 (71.22)
Size of full *yingchou* network	
> 5	207 (53.77)
≤ 5	178 (46.23)
Density of core *yingchou* network^a^	0.59 (0.34)
Participant-reported number of alters in core *yingchou* network who had commercial sex	
0	230 (59.74)
≥ 1	155 (40.26)
Participant-reported number of alters in core *yingchou* network who had commercial sex (subsample, *N* = 139)	
0	38 (27.34)
≥ 1	101 (72.66)
Participant-reported number of alters in core *yingchou* network who always used condoms in commercial sex (subsample, *N* = 139)	
0	74 (53.24)
≥ 1	65 (46.76)
Weighted commercial sex norm of core *yingchou* network^a^	−0.02 (38.66)
Weighted condom use norm of core *yingchou* network (*N* = 139)^a^	8.28 (33.94)

^a^Mean (SD)

Among the full sample, 40.3% (155/385) of participants reported at least one alter in their core *yingchou* network who had commercial sex in the last year. Among the subsample of participants who had commercial sex in the past year, 72.7% (101/139) reported at least one alter who had commercial sex, and 46.8% (65/139) reported at least one alter who always used condoms in commercial sex in the last year. The average scores of the weighted commercial sex norm and the weighted condom use norm of core *yingchou* network were −0.02 (range, −104-111; SD = 38.66) and 8.28 (range, −82-111; SD = 33.94), respectively.

### Commercial sex and *yingchou* network

Table 2 presents the results of multivariate logistic analyses of the association between commercial sex in the last year and various characteristics. Model 1 was an individual model, which included only individual-level characteristics. As revealed by Model 1, compared to participants with more than high school education, participants with no more than high school education were 3.17 (95% CI, 1.63–6.18; *p* < 0.01) times more likely to have had commercial sex in the last year. Compared to participants with a steady sex partner, participants without a steady sex partner were 3.54 (95% CI, 1.46–8.56; *p* < 0.01) times more likely to have had commercial sex in the last year. The higher the perceived social motivations of commercial sex, the more likely participants were to report commercial sex (OR, 1.61; 95% CI, 1.45–1.78; *p* < 0.001). Age was marginally significantly associated with commercial sex (OR, 0.97; 95% CI, 0.93–1.00; *p* < 0.10).

**Table 2:** Logistic analysis of commercial sex in the last year (*N* = 385)

Characteristic (reference)	Commercial sex in the last year odds ratio (95% confidence interval)		
	Model 1	Model 2	Model 3
Age	0.97 (0.93–1.00)†	0.96 (0.92–1.01)†	0.95 (0.91–1.00)*
≤ High school (> high school)	3.17 (1.63–6.18)**	3.95 (1.83–8.56)***	4.19 (1.84–9.53)**
≥ 5000 yuan (< 5000 yuan)	1.50 (0.76–2.95)	1.78 (0.80–3.97)	1.65 (0.72–3.82)
Self-employed or else (employed)	0.83 (0.45–1.51)	1.19 (0.59–2.39)	1.25 (0.60–2.60)
Unmarried or else (married)	0.74 (0.32–1.67)	0.76 (0.29–1.99)	0.86 (0.31–2.50)
No steady sex partner (yes)	3.54 (1.46–8.56)**	5.45 (1.91–15.59)**	5.08 (1.71–15.06)**
≥ *Yingchou* twice per month (< twice per month)	1.45 (0.79–2.65)	1.54 (0.75–3.17)	1.76 (0.78–3.58)
Perceived vulnerability to HIV/STIs	0.95 (0.85–1.07)	0.90 (0.78–1.03)	0.90 (0.77–1.04)
Perceived severity of being infected with HIV/STIs	0.83 (0.64–1.07)	1.00 (0.75–1.34)	1.04 (0.78–1.39)
Perceived necessity & effectiveness of condom use in commercial sex	1.13 (0.93–1.36)	1.05 (0.86–1.28)	1.08 (0.87–1.34)
Perceived social motivations of commercial sex	1.60 (1.45–1.78)***	1.54 (1.36–1.76)***	1.62 (1.40–1.87)***
Size of *yingchou* network >5 (≤ 5)		0.55 (0.27–1.12)†	0.50 (0.24–1.08)†
Density of core *yingchou* network		1.46 (0.52–4.11)	1.19 (0.40–3.54)
Alters having commercial sex ≥1 (0)		5.67 (2.75–11.67)***	
Weighted commercial sex norm of core *yingchou* network			1.03 (1.02–1.04)***
Model G^2^ (−2 Loglikelihood)	308.96	232.97	208.40
Degrees of freedom	11	14	14
Changed Chi square		75.99***	100.56***

†*p* < .10, *p < .05, **p < .01, ***p < 001

Model 2 added three network variables to Model 1 (the size of full *yingchou* network, the density of core *yingchou* network, and the number of alters having commercial sex in the core *yingchou* network). The results showed that the formerly significant individual variables remained significant. The size of the full *yingchou* network was only marginally significantly associated with commercial sex and the density of the core *yingchou* network was not significant. The number of alters having commercial sex in the core *yingchou* network was significantly associated with participants’ commercial sex. Compared to participants with no alter who had participated in commercial sex, participants with one or more such alters were 5.67 times more likely to have participated in commercial sex in the last year (95% CI, 2.75–11.67).

Model 3 used the weighted measure of commercial sex norms of the core *yingchou* network which integrated both the measure of uncertainty when egos reported alters’ commercial sex behavior and the intimacy of alter-ego ties. As illustrated in Table 2, with each one-point increase in the commercial sex norm score (range, −104-111), participants were 1.03 times more likely to have commercial sex in the last year (95% CI, 1.02–1.04). The size of the full *yingchou* network was still marginally significant (*p* < 0.10) in Model 3, and participants’ age was significant (*p* < 0.05), with older participants less likely to report commercial sex in the past year.

The Chi square analysis of changes in Model G^2^ revealed that the inclusion of network variables significantly improved the goodness-of-fit of Model 2 and Model 3 as compared to Model 1 (*P* < 0.001). Compared to Model 2, Model 3 had similar degrees of freedom but a larger change in *X* ^*2*^, hence a better-fitting model. However, it could not be determined whether the difference between Model 2 and Model 3 was significant because they were not nested.

### Condom use in commercial sex and *yingchou* network

The results of three logistic models of condom use in commercial sex are presented in Table 3. Among individual characteristics, only two perception factors were consistently significant (*p* < 0.001) across all three models. The higher the perceived severity of being infected with HIV/STIs, the more likely participants were to report consistent condom use in commercial sex in the last year. The higher participants perceived their vulnerability to HIV/STIs to be, the less likely they were to consistently use condoms in commercial sex in the last year. Frequency of participating in *yingchou* activities was only significantly associated with consistent condom use in Models 1 and 3.

**Table 3:** Logistic analysis of condom use in commercial sex in the last year (*N* = 139)

Characteristic (reference)	Consistent condom use in commercial sex odds ratio (95% confidence interval)		
	Model 1	Model 2	Model 3
Age	0.99 (0.92–1.07)	1.01 (0.91–1.11)	1.04 (0.93–1.16)
≤ High school (> high school)	0.81 (0.24–2.76)	0.73 (0.16–3.31)	0.54 (0.12–2.51)
≥ 5000 yuan (< 5000 yuan)	2.22 (0.68–7.20)	1.18 (0.27–5.15)	1.00 (0.20–4.90)
Self-employed or else (employed)	1.79 (0.53–6.07)	2.98 (0.69–12.91)	2.09 (0.47–9.19)
Unmarried or else (married)	0.81 (0.20–3.32)	2.06 (0.30–14.37)	1.67 (0.23–11.24)
No steady sex partner (yes)	0.83 (0.19–3.56)	0.55 (0.09–3.49)	0.67 (0.12–3.93)
≥ *Yingchou* twice per month (< twice per month)	3.70 (1.33–10.28)*	3.58 (0.88–14.49)†	4.74 (1.14–19.75)*
Perceived vulnerability to HIV/STIs	0.71 (0.59–0.86)***	0.59 (0.44–0.79)***	0.60 (0.45–0.80)***
Perceived severity of being infected with HIV/STIs	1.76 (1.08–2.87)*	2.33 (1.21–4.49)*	2.09 (1.15–3.81)*
Perceived necessity & effectiveness of condom use in commercial sex	1.43 (1.00–2.06)†	0.95 (0.59–1.55)	1.11 (0.68–1.79)
Size of *yingchou* network >5 (≤ 5)		1.91 (0.45–8.10)	2.84 (0.58–14.06)
Density of core *yingchou* network		1.34 (0.18–10.04)	1.55 (0.17–14.25)
Alters always using condoms in commercial sex ≥1 (0)		11.95 (2.42–59.01)**	
Weighted condom use norm of core *yingchou* network			1.04 (1.02–1.07)**
Model G^2^ (−2 Loglikelihood)	114.29	76.96	72.21
Degrees of freedom	10	13	13
Changed Chi square		37.33***	42.08***

†*p* < .10, *p < .05, **p < .01, ***p < .001

Both measures of condom use norms of the core *yingchou* network were significantly associated with participants’ consistent condom use in commercial sex. As revealed in Model 2, compared to participants with no alters who always used condoms in commercial sex, participants with one or more such alter were 11.95 (95% CI, 2.42–59.01) times more likely to use condoms consistently in commercial sex. Model 3 showed that with each one-point increase in the condom use norms score, participants were 1.04 times more likely to consistently use condoms in commercial sex (95% CI, 1.02–1.07).

As shown by the Chi square/G^2^ statistics, both network models were significantly better-fitting models (*p* < 0.001) than the individual model. Among the network models, Model 3 had a smaller G^2^ with similar degrees of freedom, which suggested that the fit of Model 3 was better than that of Model 2.

## Discussion

In this sample of rural-to-urban male migrants in China, the norms of core *yingchou* networks (measured in two different ways) were significantly associated with egos’ sexual risk behaviors. Participants who had a more pro-commercial sex and pro-condom use core *yingchou* network were significantly more likely to have commercial sex in the last year and consistently use condoms in commercial sex, respectively. Furthermore, as compared to an individual-level model, the inclusion of social network variables significantly improved the fit of the models predicting commercial sex and condom use. As discussed below, the unique contributions of this study are threefold: applying a social network approach to HIV risk among male migrants in China; identifying a specific social network, that is, the core *yingchou* network, that is relevant for HIV-related risk and protective behaviors among this subpopulation; and demonstrating the importance of measuring the intimacy of ego-alter ties when studying social network norms.

This study is consistent with past studies on HIV risk which showed that social networks exerted strong influence on the behavior of network members. Among a sample of homeless men who have sex with men in the U.S., having social network members who regularly attended school and did not drink alcohol heavily predicted significantly lower likelihood of engaging in high-risk sex [36]. Similarly, a study among male clients of commercial sex in China found that consistent condom use with female sex workers was significantly more likely among male clients who perceived more pro-condom norms among their peer groups [29]. Studies conducted among rural-to-urban migrants in China also revealed the role of social networks in shaping members’ attitudes. For example, migrants’ attitudes towards premarital and extramarital sex were found significantly associated with the corresponding attitudes of their network members [20]. While the importance of social networks has been recognized in both HIV research and China migrant studies, a social network approach has not been applied to their intersection, that is, studies on HIV risk among rural-to-urban migrants in China. The present study thus extends prior understanding of the role of social networks in shaping risk behaviors within this substantial population.

The second contribution of this study lies in the identification of a specific type of social network, that is, the core *yingchou* network, that has implications for HIV-risk and protective behaviors among male migrants in China. In this study, we drew on former studies of *yingchou* activities in China which clearly showed the very common involvement of commercial sex in this gendered cultural practice for network building [24–28]. While former studies, which were mainly qualitative, viewed *yingchou* from a perspective of dynamic networking process, this study viewed it from a structural perspective which highlighted the network resulting from the networking process. As we hypothesized, the networks that resulted from *yingchou* activities were significantly associated with male migrants’ commercial sex and condom use. Our results also suggest the relevance of exploring other potentially important social network structures in future studies. For example, the networks of hometown fellows (*laoxiang*) have been described as important social resources for migrants who were seeking jobs (including commercial sex), conducting business, exchanging rental information, and acquiring low interest rate loans in cities [37–40]. Future work could explore whether and how these *laoxiang* networks shape sexual risk behaviors among migrants and the potential overlap between *laoxiang* and *yingchou* networks.

The third contribution of the current study is the demonstration of a more sensitive network model which takes into account the intimacy of network ties and allows for egos’ uncertainty when reporting their alters’ sexual behaviors. This approach is based on and consistent with the long recognized fact that social influence is not evenly distributed within a social network [41, 42] and the newer evidence that strong ties (greater relationship intimacy) are more influential in affecting behavior changes [43–46]. Future studies should continue to consider the nuanced influences within social networks, such as the strength of social ties.

Interestingly, other network characteristics including the size and density of *yingchou* networks had weaker relationships with egos' HIV risk behaviors. Specifically, in the two network-based models, we found that the size of the *yingchou* network was only marginally significantly associated with commercial sex. This result is not consistent with a former study showing that the size of marriage discussion network was significantly associated with migrants’ attitudes to pre- and extra-marital sex [20]. The density of core *yingchou* network was also not significantly associated with egos’ behaviors, which is consistent with findings from another study conducted among injection drug users in the U.S. [17]. While these findings may suggest the different influences of various network characteristics on a behavior, it should be noted that the non-significance of network size and density indicators could also be attributed to measurement challenges. Since participants in this study were asked to recall the number of people who networked with them in one year, their answers were vulnerable to recall bias. In addition, the imposed three-alter limit of the core *yingchou* network might not have allowed sufficient heterogeneity of network density to emerge. Network size and density variables warrant more attention from future network studies on HIV risk among internal migrants in China.

Our findings suggest several implications for the development of HIV prevention interventions. First, the relevance of core *yingchou* network to commercial sex and condom use warrants a network intervention among male migrants in China. Former studies conducted among drug users showed that network-based interventions were significantly associated with reductions in HIV-related risk [47–49]. Similar to drug use behaviors, commercial sex in China is deemed morally deviant, which makes it very difficult for male clients of commercial sex to share their commercial sex and condom use experiences with people other than members of a small group who networked and visited female sex workers together [27, 28]. Network-based peer interventions hold promise compared to more traditional media channels [50] and may be particularly useful for disseminating sexual health information to exclusive, hard-to-reach networks – such as *yingchou* networks. Since traditional intervention models that utilize peer educators or social networks, such as the Community Popular Opinion Leader model and Peer Health Educator model, tend to be based on geographically set locations and settings, they may neglect the importance of social ties outside of specific settings and the role of cross-setting social networks [51]. In this study, core *yingchou* networks were theoretically cross-setting social networks because of the employment of the ego-centric approach and the open choices in reporting alters. Thus, the relevance of core *yingchou* network to HIV risk reveals the need in the future to consider cross-setting social networks in conducting network-oriented HIV prevention and intervention. Furthermore, as China is marching into an era of social media [52], it is also relevant to ask how network-oriented HIV interventions may be promoted via popular social media platforms [53, 54].

There were several limitations to this study. First, a cross-sectional design limited our ability to make a causal inference of the relationship between network variables and egos’ behaviors. Second, a convenience sample might limit the generalizability of our findings. However, we employed two measures to increase the heterogeneity of the sample. First, we did not use referrals or snowball sampling which might increase the homogeneity of the sample. Second, we sampled participants from diverse recruitment settings which resulted in greater heterogeneity across demographics and socioeconomic strata of migrants. Future studies employing a random sampling are necessarily called for to verify the results of this study. Finally, a three-alter limit set for the core *yingchou* network might have not given enough space for network heterogeneity to emerge.

## Conclusions

This study is one of the first to apply a social network approach to the study of HIV risk among internal migrants in China. By showing clearly the relevance of social networks to a better understanding of HIV risk and protective behaviors, this study calls for a “network turn” in both the study and prevention practice of HIV risk among rural-to-urban migrants in China. Furthermore, greater attention in social network studies is warranted around the measurement and analysis of the differential influence of specific network characteristics on the outcomes of interest. Understanding the important roles *yingchou* networks play in shaping HIV risk among male migrants in China is a critical step toward designing effective social network-based HIV prevention interventions.

## References

[1] Qiao, X. An analysis of internal migrants based on the Fifth National Population Census of China. Sociol Stud (China). 2003;1:87–94.

[2] National Health and Family Planning Committee of China. Report on 2015 China's migration population development http://www.moh.gov.cn/xcs/s3574/201511/07b8efe0246e4a59bd45d1fd7f4e3354.shtml. Accessed 20 Feb 2016.

[3] Brockerhoff M, Biddlecom AE (1999). Migration, sexual behavior and the risk of HIV in Kenya. Int Migr Rev.

[4] Jochelson K, Mothibeli M, Leger J (1991). Human immunodeficiency virus and migrant labor in South Africa. Int J Health Serv.

[5] Organista KC, Organista PB (1997). Migrant laborers and AIDS in the United States: a review of the literature. AIDS Educ Prev.

[6] Li X, Zhang L, Stanton B, Fang X, Xiong Q, Lin D (2007). HIV/AIDS-related sexual risk behaviors among rural residents in China: potential role of rural-to-urban migration. AIDS Educ Prev.

[7] Zhang L, Chow EPF, Jahn HJ, Kraemer A, Wilson DP (2013). High HIV prevalence and risk of infection among rural-to-urban migrants in various migration stages in China: a systematic review and meta-analysis. Sex Trans Dis.

[8] Zou X, Chow EPF, Zhao P, Xu Y, Ling L, Zhang L (2014). Rural-to-urban migrants are at high risk of sexually transmitted and viral hepatitis infections in China: a systematic review and meta-analysis. BMC Infect Dis.

[9] Zhuang X, Wu Z, Poundstone K, Yang C, Zhong Y, Jiang S. HIV-related high-risk behaviors among Chinese migrant construction laborers in Nantong, Jiangsu. PLoS One. 2012;7(3): e31986 Epub 2012 Mar 30. 10.1371/journal.pone.0031986 PMC3316523 22479313

[10] Li X, Fang X, Lin D, Mao R, Wang J, Cottrell L, Harris C, Stanton B (2004). HIV/STD risk behaviors and perceptions among rural–to–urban migrants in China. AIDS Educ Prev.

[11] Lin D, Li X, Yang H (2005). Alcohol intoxication and sexual risk behaviors among rural-to-urban migrants in China. Drug Alcohol Depend.

[12] Smith CJ (2005). Social geography of sexually transmitted diseases in China: exploring the role of migration and urbanization. Asia Pac Viewp.

[13] Yang X. Temporary migration and the spread of STDs/HIV in China: is there a link?Int Migr Rev. 2004; 38(1):212–35.

[14] Yang X, Derlega VJ, Luo H (2007). Migration, behaviour change and HIV/STD risks in China. AIDS Care.

[15] Friedman S, Aral S (2001). Social networks, risk-potential networks, health, and disease. J Urban Health.

[16] Rhodes T, Singer M, Bourgois P, Friedman SR, Strathdee SA (2005). The social structural production of HIV risk among injecting drug users. Soc Sci Med.

[17] Latkin CA, Kuramoto SJ, Davey-Rothwell MA, Tobin KE (2010). Social norms, social networks, and HIV risk behavior among injection drug users. AIDS Behav.

[18] Li P (1996). The social network and social status of migrant workers. Sociol Stud (China).

[19] Li S, Yang S, Ren Y, Jin X (2007). Social network of rural-urban migrants and their occupation stratum and income-findings from survey in Shenzhen. Mod Econ Sci (China).

[20] Jin X, Ren F, Yue Z (2008). Rural migrant workers attitudes towards premarital and extramarital sexual behaviors: a study of social networks. Popul Stud (China).

[21] Guo Y, Li X, Fang X, Lin X, Song Y, Jiang S, Stanton B (2011). A comparison of four sampling methods among men having sex with men in China: implications for HIV/STD surveillance and prevention. AIDS Care.

[22] Wang B, Li X, Stanton B, Liu Y, Jiang S (2013). Socio-demographic and behavioral correlates for HIV and syphilis infections among migrant men who have sex with men in Beijing. China. AIDS Care..

[23] Laumann E, Marsden P, Prensky D, Burt RS, Minor MJ (1983). The boundary specification problem in network analysis. Applied network analysis: a methodological introduction.

[24] Zheng T (2012). Entrepreneurial masculinity, health, and the state in post-socialist China. Int J Mens Health.

[25] Zheng T (2006). Cool masculinity: male clients' sex consumption and business alliance in urban China's sex industry. J Contemp China.

[26] Uretsky E (2008). ‘mobile men with money’: the socio-cultural and politico-economic context of ‘high-risk’ behaviour among wealthy businessmen and government officials in urban China. Cult Health Sex.

[27] Wang W, Muessig KE, Li M, Zhang Y (2014). Networking activities and perceptions of HIV risk among male migrant market vendors in China. AIDS Behav.

[28] Yang C, Latkin CA, Liu P, Nelson KE, Wang C, Luan R (2010). A qualitative study on commercial sex behaviors among male clients in Sichuan Province. China AIDS Care.

[29] Yang C, Latkin C, Luan R, Nelson K (2010). Peer norms and consistent condom use with female sex workers among male clients in Sichuan Province. China Soc Sci Med.

[30] van Gennep A (1960). The rites of passage.

[31] Wasserman S, Faust K (1994). Social network analysis: methods and applications.

[32] Chorography Compile Committee of Beijing City. Beijing Chronicle 2014. http://www.bjsfzg.org.cn/read/ISSN1002-36582014. Accessed 20 Feb 2016.

[33] Burt R (1984). Network items and the general social survey. Soc Networks.

[34] Cornwell B, Schumm LP, Laumann EO, Graber J (2009). Social network in the NSHAP study: rationale, measurement, and preliminary findings. J Gerontol B Psychol Sci Soc Sci.

[35] Power DA, Xie Y (1999). Statistical methods for categorical data analysis.

[36] Tucker JS, Hu J, Golinelli D, Kennedy DP, Green HD, Wenzel SL (2012). Social network and individual correlates of sexual risk behavior among homeless young men who have sex with men. J Adolesc Health.

[37] Fan CC (2002). The elite, the natives, and the outsiders: migration and labor market segmentation in urban China. Ann Assoc Am Geogr.

[38] Mobrand E (2006). Politics of cityward migration: an overview of China in comparative perspective. Habitat Int.

[39] Wu W (2006). Migrant intra-urban residential mobility in urban China. Hous Stud.

[40] Xiang B (2004). Transcending boundaries: Zhejiangcun: the story of a migrant village in Beijing.

[41] Katz E, Lazarsfeld PF (1955). Personal influence: the part played by people in the flow of mass communications.

[42] Rogers EM (1962). Diffusion of innovations.

[43] Christakis NA, Fowler JH (2007). The spread of obesity in a large social network over 32 years. The N Engl J Med.

[44] Christakis NA, Fowler JH (2008). The collective dynamics of smoking in a large social network. N Engl J Med.

[45] Goldsmith EB (2015). Social influence and sustainable consumption.

[46] Bond RM, Fariss CJ, Jones JJ, Kramer ADI, Marlow C, Settle JE, Fowler JH (2012). A 61-million-person experiment in social influence and political mobilization. Nature.

[47] Latkin CA, Donnell D, Metzger D, Sherman S, Aramrattna A (2009). Davis- Vogel a, Quan VM, Gandham S, Vongchak T, Perdue T, Celentano DD. The efficacy of a network intervention to reduce HIV risk behaviors among drug users and risk partners in Chiang Mai, Thai- land and Philadelphia, USA. Soc Sci Med.

[48] Sherman SG, Sutcliffe C, Srirojn B, Latkin CA, Aramratanna A, Celentano DD (2009). Evaluation of a peer network intervention trial among young methamphetamine users in Chiang Mai. Thailand Soc Sci Med.

[49] Hoffman IF, Latkin CA, Kukhareva PV, Malov SV, Batluk JV, Shaboltas AV, Skochilov RV, Sokolov NV, Verevochkin SV, Hudgens MG, Kozlov AP (2013). A peer-educator network HIV prevention intervention among injection drug users: results of a randomized controlled trial in St. Petersburg, Russia. AIDS Behav.

[50] Youm Y, Laumann EO (2002). Social network effects on the transmission of sexually transmitted diseases. Sex Transm Dis.

[51] Schneider J. Kumar SP. Towards Social network HIV prevention interventions in migrant workers. https://www.researchgate.net/publication/242586523_Towards_Social_Network_HIV_Prevention_Interventions_in_Migrant_Workers. Accessed 21 Oct 2016

[52] Chiu C, Ip C, Silverman A (2012). Understanding social media in China. McKinsey Quarterly.

[53] Garett R, Smith J, Young SD (2016). A review of social media technologies across the global HIV care continuum. Curr Opin Psychol.

[54] Tso LS, Tang W, Li H, Yan HY, Tucker JD (2016). Social media interventions to prevent HIV: a review of interventions and methodological considerations. Curr Opin Psychol..

